# Visualization of Hg^2+^ Stress on Plant Health at the Subcellular Level Revealed by a Highly Sensitive Fluorescent Sensor

**DOI:** 10.34133/research.0570

**Published:** 2025-01-07

**Authors:** Sumeera Asghar, Zhenyang Yu, Zheng Zhu, Dengyue Zheng, Zimo Zhao, Yuming Xu, Xiao Liu, Chao Yuan, Yan Li, Wei Wang, Jianfeng Xu, Huailong Teng, Jun Li, Wen-Chao Yang, Chunli Chen

**Affiliations:** ^1^ State Key Laboratory of Green Pesticide, Key Laboratory of Green Pesticide and Agricultural Bioengineering, Ministry of Education, Center for R&D of Fine Chemicals of Guizhou University, Guiyang 550025, China.; ^2^ The Key Laboratory of Plant Resources Conservation Germplasm Innovation in Mountainous Region, College of Life Sciences, Institute of Agro-bioengineering, Guizhou University, Guiyang 550025, China.; ^3^ Hubei Hongshan Laboratory, College of Life Science and Technology, Huazhong Agricultural University, Wuhan, Hubei 430070, China.; ^4^ National Key Laboratory for Germplasm Innovation and Utilization for Fruit and Vegetable Horticultural Crops, Wuhan, China.; ^5^ College of Chemistry, Huazhong Agricultural University, Wuhan, Hubei 430070, China.; ^6^ School of Environmental Science and Engineering, Guangdong University of Petrochemical Technology, Maoming 525000, China.

## Abstract

The presence of Hg^2+^ causes substantial stress to plants, adversely affecting growth and health by disrupting cell cycle divisions, photosynthesis, and ionic homeostasis. Accurate visualization of the spatiotemporal distribution of Hg^2+^ in plant tissues is crucial for the management of Hg pollution; however, the related research is still at its early stage. Herein, a small-molecule amphiphilic fluorescent probe (termed **LJTP2**) was developed for the specific detection of Hg^2+^ with a high sensitivity (~16 nM). Fluorescent imaging applications with **LJTP2** not only detected the dynamic distribution of Hg^2+^ within plant cells at the subcellular level but also enabled the understanding of cell membrane health under Hg^2+^ stress. This study introduces a valuable imaging tool for elucidating the molecular mechanism of Hg^2+^ stress in plants, demonstrating the potential of the application of small-molecule fluorescent probes in plant science.

## Introduction

Heavy metal pollution, which is nonbiodegradable and accumulates in soils through anthropogenic activities, not only affects plant growth and crop yield via complicated mechanisms in agriculture but also induces health problems through food chains. As a mainly toxic species of heavy metal, mercury (Hg) pollution poses a global concern, stemming from both natural phenomena and human activities [1–3]. As a major form of Hg, Hg^2+^ is absorbed by plant roots and transferred to the leaves via a transport system, which could further induce severe phytotoxicity and impair plant metabolic processes and growth [4]. Rice, one of the major food sources, played an important role in the survival of human beings and social stability. However, the long-term accumulation of Hg^2+^ in rice can induce serious issues, including food and ecological safety, human health, and crop yield [5–7].

Effective management of Hg pollution in modern agriculture requires an accurate assessment of Hg^2+^ distribution in plant tissues and the damage it causes at the subcellular level [8,9]. Among various detection tools, such as atomic absorption/emission spectroscopy, inductively coupled plasma mass spectrometry, and isotopic labeling [10], fluorescent sensors have displayed marked superiorities, including high sensitivity and spatiotemporal resolution and noninvasive in situ imaging [11–23]. So far, some fluorescent sensors have been developed for in vivo detection of Hg^2+^ [24–39]*.* For example, Chen et al. [33] developed a boron-dipyrromethene-based fluorescent probe for Hg^2+^ imaging in *Arabidopsis* with near-infrared emission. Wang et al.’s group [29] developed a coumarin-based fluorescent probe based on the mechanism of Hg^2+^-induced desulfurization. The probe was successfully applied for fluorescent imaging in *Arabidopsis* root. Recently, An and co-workers designed a fluorescent probe based on aggregation-induced emission enhancement for Hg^2+^ detection. The probe can be employed to detect Hg^2+^ in the rhizome slices of Radix Hedysari under an ultraviolet lamp [39]. Despite great progress achieved in plant imaging, there is still an urgent need to design an efficient probe that can be used to evaluate Hg^2+^-induced damage against plant cells at the subcellular level, which is crucial for managing Hg pollution.

**Fig. 1.**
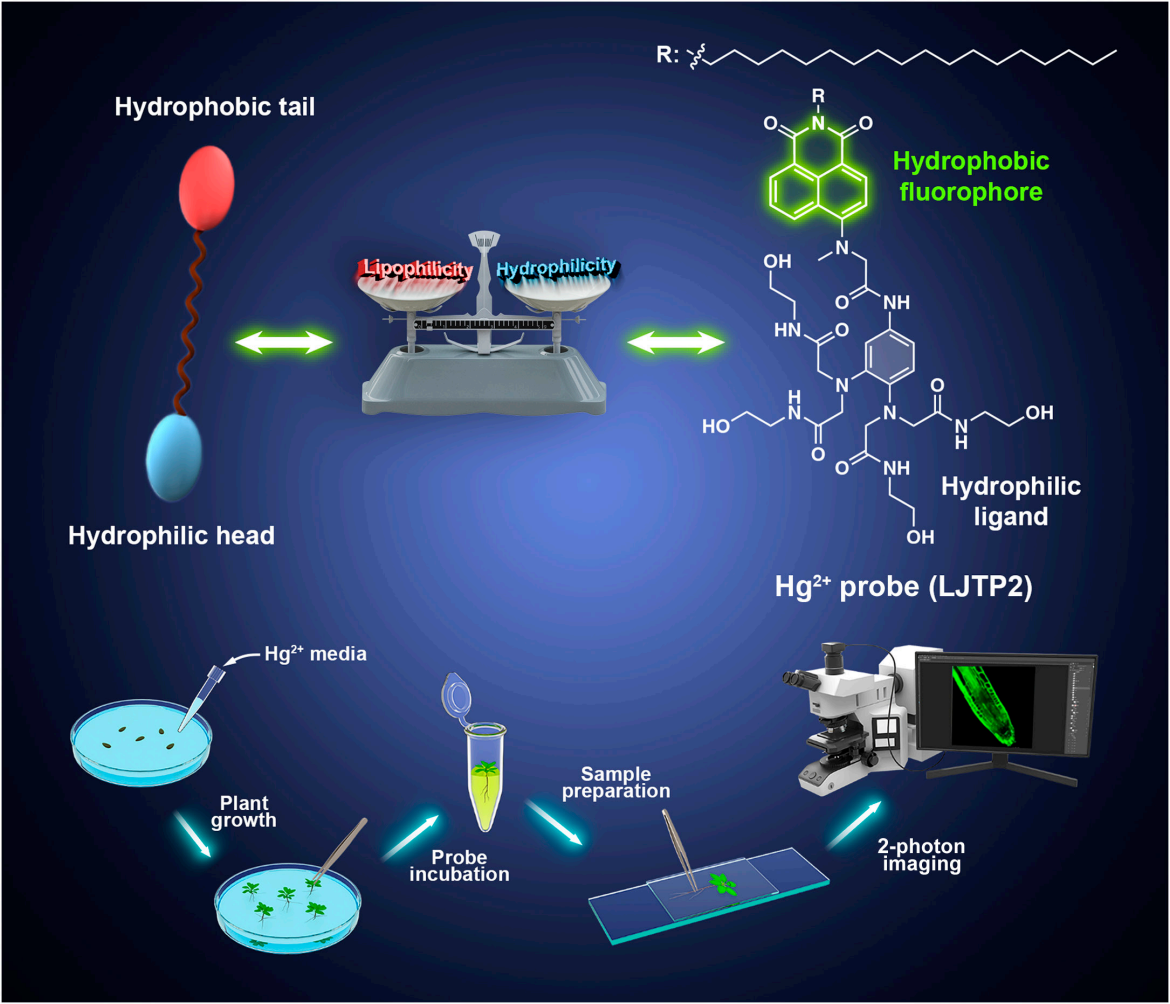
The design strategy of the fluorescent probe (LJTP2) for detection and imaging Hg^2+^ in plant tissues.

In this study, a novel fluorescent probe (**LJTP2**) was synthesized to visualize the subcellular distribution and translocation of Hg^2+^ in plant tissues. **LJTP2** simulated the amphiphilic nature of surfactants, which improved the probe’s dispersibility in aqueous solution. It comprises a lipophilic octadecyl group enabling the probe to target cells, a naphthylamide-based fluorophore for signal output, and a hydrophilic tetrakis (*N*-2-hydroxyethyl) acetamide group as a Hg^2+^-specific binding component (Fig. 1 and Fig. S11). Due to its amphipathic property, **LJTP2** not only showed good selectivity but also displayed an ultralow limit of detection (LOD) of 16 nM toward Hg^2+^ early detection due to its high sensitivity. The fluorescent probe signals were observed for Hg^2+^ detection in the model plant *Arabidopsis* (root tip and leaf), moss (another model plant, an important plant in terms of evolutionary studies), and an onion to visualize its location and further its penetration at the subcellular level. More importantly, the Hg^2+^ distribution and its damage to plant cells were clearly observed under single- and 2-photon microscopy.

## Results

### Design and optical characterization of the developed sensor

**Fig. 2.**
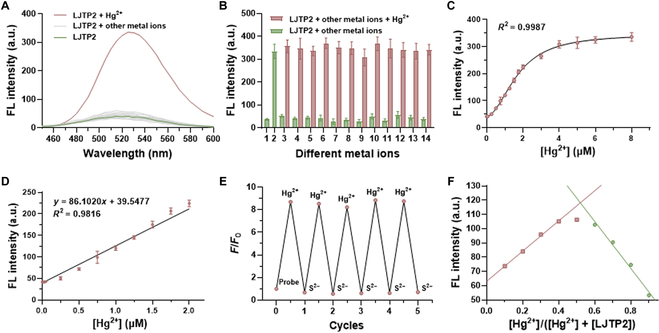
The optical properties of LJTP2. (A) Fluorescence response of LJTP2 (10 μM) toward various metal ions (100 μM). (B) Fluorescence selectivity of LJTP2 with Hg^2+^ in the presence of various metal ions, including 1, probe only; 2, Hg^2+^; 3, Mg^2+^; 4, Fe^3+^; 5, Al^3+^; 6, Na^+^; 7, Cu^2+^; 8, Fe^2+^; 9, Ag^+^; 10, Pd^2+^; 11, Mn^2+^; 12, Cd^2+^; 13, Zn^2+^; and 14, Li^+^. (C) Fluorescence titration of LJTP2 (10 μM) with different concentrations of Hg^2+^. (D) Linear relationship of LJTP2 with difference concentrations of Hg^2+^ in the range of 0 to 2.0 μM. (E) Fluorescence response of LJTP2 based on emission at 525 nm in cycles of Hg^2+^ (10 μM) addition and subsequent Na_2_S (10 μM) treatment. (F) Job plots of the fluorescence intensity of LJTP2 as a function of Hg^2+^ concentration. FL, fluorescence.

With the probe in hand, we first evaluated the probe’s optical properties in aqueous solution. **LJTP2** showed a strong absorption peak at 410 nm in Hepes solution (Fig. S12), which was employed as the excitation wavelength for fluorescence measurement. The fluorescence titration experiment of **LJTP2** with different metal ions, including Fe^3+^, Al^3+^, Fe^2+^, Zn^2+^, Cd^2+^, Hg^2+^, Mg^2+^, Cu^2+^, Pd^2+^, Mn^2+^, Li^+^, Na^+^, and Ag^+^, showed that only Hg^2+^ induced significant fluorescence enhancement at an emission peak of 525 nm, indicating the good selectivity of **LJTP2** (Fig. 2A and B). A good linear relationship between the enhancement of fluorescence intensity and the concentration of Hg^2+^ can be found within a range of 0 to 3 μM (Fig. 2C and D), and the LOD was determined as 16 nM. Due to its special affinity toward S^2−^, the Hg^2+^-enhanced fluorescence could be recovered back to that of the free probe. This rapidly reversible sensing behavior is repeated 5 times without obvious signal attenuation (Fig. 2E). To understand the binding mode of **LJTP2** with Hg^2+^, a Job plot experiment was performed. As shown in Fig. 2F, the intersection of the curve was located at a ratio of 0.5, indicating the 1:1 binding ratio of **LJTP2** with Hg^2+^ (Fig. S11). Furthermore, **LJTP2** was very stable in the pH range of 6.5 to 8.5, which can be used for Hg^2+^ sensing under physiological conditions (Fig. S13).

### Density functional theory calculations

**Fig. 3.**
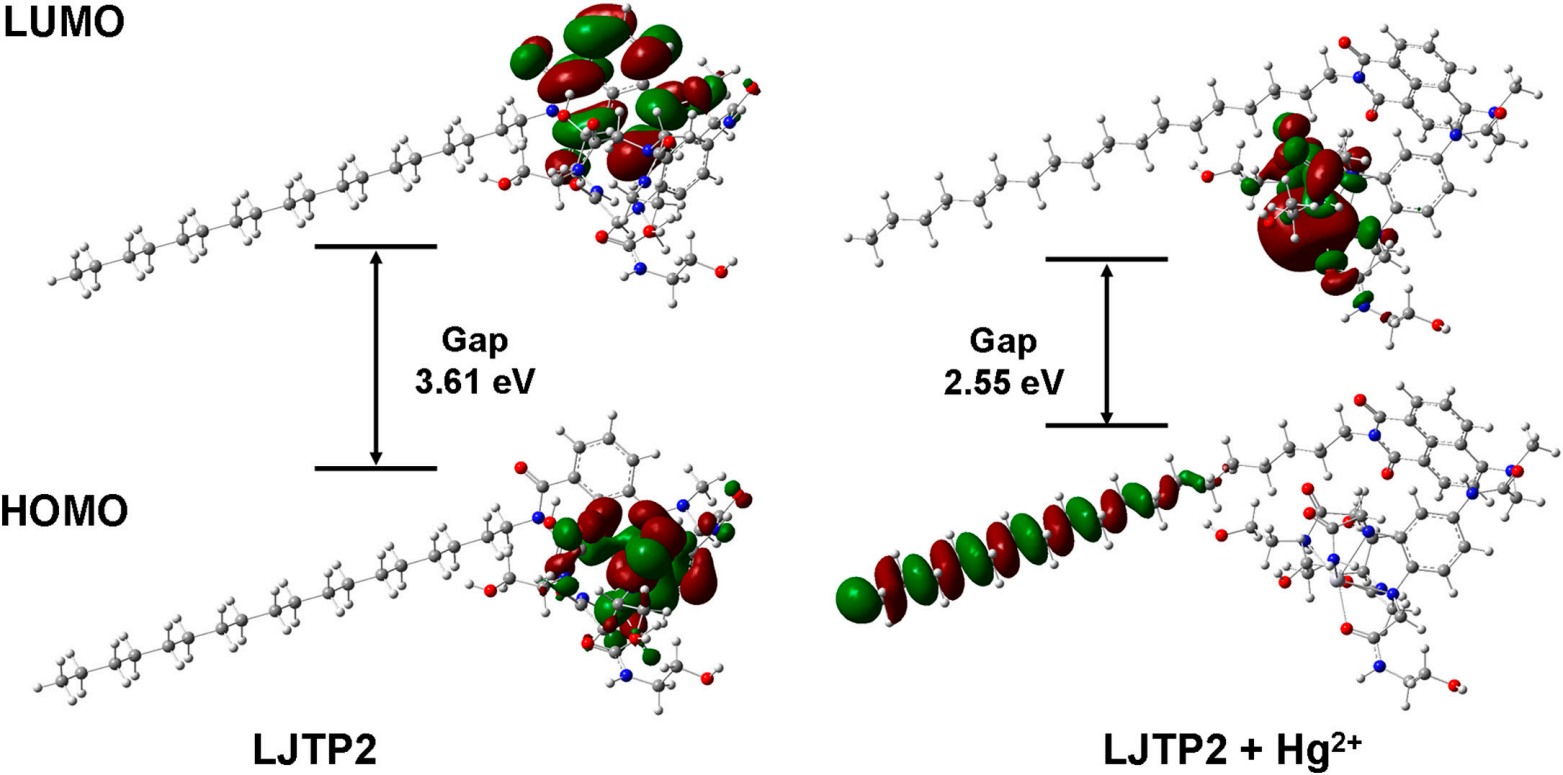
Molecular orbitals and corresponding energy levels of LJTP2 and LJTP2 + Hg^2+^ in both the ground state and excitation state. LUMO, lowest unoccupied molecular orbital; HOMO, highest occupied molecular orbital.

To understand the sensing mechanism (Fig. S11), a density functional theory calculation was conducted using the Gaussian 16 software [40]. The highest occupied molecular orbital and lowest unoccupied molecular orbital were distributed in the phenyl group and fluorophore (naphthalimide), respectively, with an energy gap of 3.61 eV; the photoinduced electron transfer occurred from the phenyl ring to the fluorophore, resulting in fluorescence off. After binding with Hg^2+^, the distribution of the highest occupied molecular orbital and lowest unoccupied molecular orbital was moved to the alkyl chain and ligand, respectively, with an energy gap of 2.55 eV, leading to the photoinduced electron transfer inhibition and fluorescence on (Fig. 3).

### In vivo imaging of Hg^2+^

**Fig. 4.**
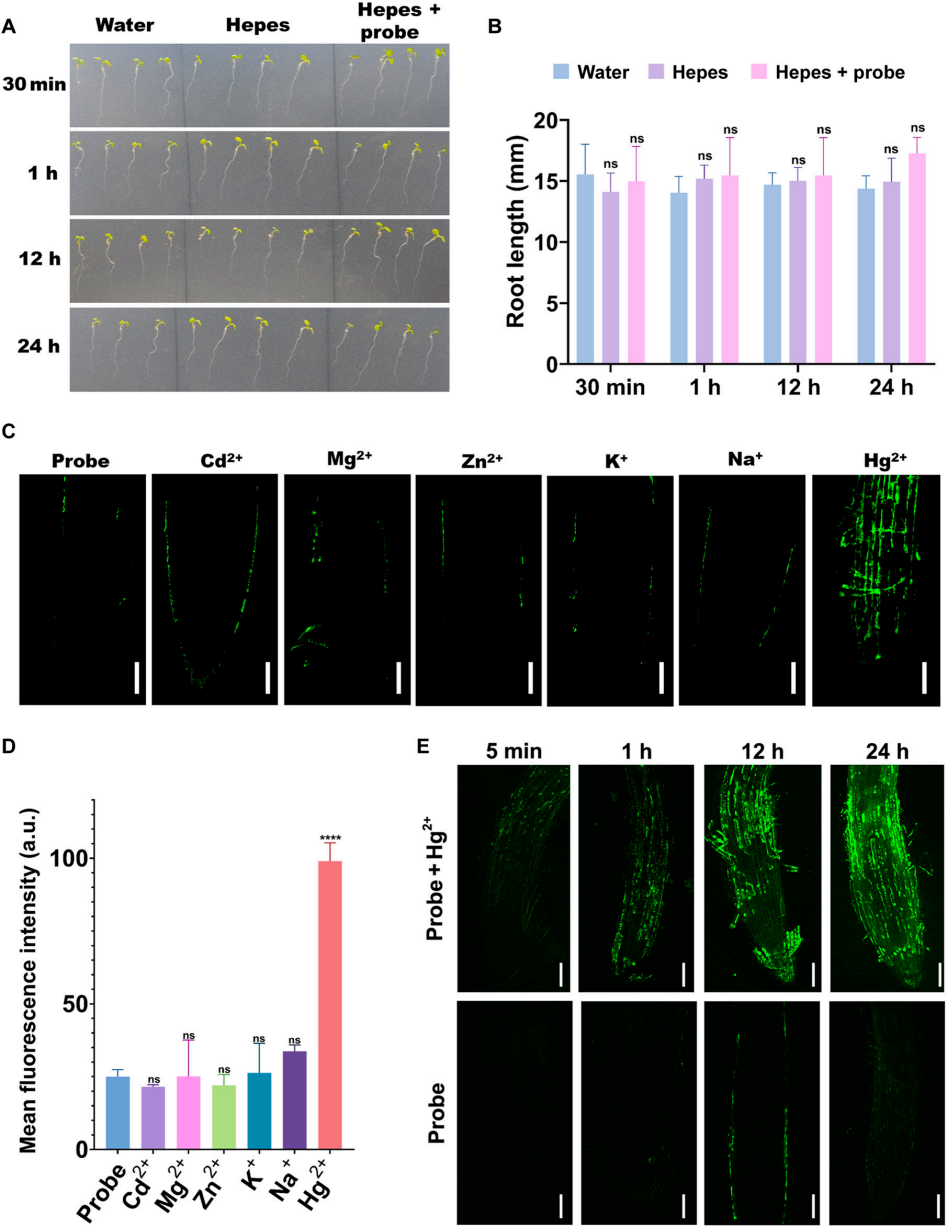
(A and B) Toxicity evaluation on plant growth after incubation with water, Hepes, and Hepes + probe. (A) Phenotypic analysis of plant growth at different times of incubation. (B) The measurement of root length at different times of incubation. (C and D) The probe’s specificity determination for different ions in plants under 2-photon microscopy (*λ*_ex_ = 750 nm); the statistical analysis of the light intensities is presented as well. ****P value ≤0.0001. (E) Assessment of the working probe solution stability over a long course of time in plant roots (scale bar = 50 μm). ns, not significant.

The probe’s biocompatibility is crucial for its biological applications; we first evaluated its toxicity on plant growth. The result showed that root length was not affected by Hepes solution or the probe working solution from a time range of 30 min until 24 h (Fig. 4A). A statistical representation of root length data under 3 different growth conditions (water, Hepes, and Hepes plus **LJTP2**) showed no side effects on root length (Fig. 4B), so it was evident that **LJTP2** did not interfere with or change the plant growth pattern. After carefully testing the probe’s toxicity toward plant growth, we went on further to explore probe specificity in vivo; the treatment of Cd^2+^, Mg^2+^, Zn^2+^, K^+^, and Na^+^ in *Arabidopsis thaliana* did not show significant fluorescent enhancement under 2-photon microscopy except Hg^2+^, and the statistically calculated mean fluorescence intensity is presented as well (Fig. 4C and D). In addition, **LJTP2** showed good signal stability in the presence and absence of Hg^2+^. The fluorescent intensity in *A. thaliana* root increased steadily after treatment with Hg^2+^ and **LJTP2** for 24 h, while no significant fluorescent signal was observed in the **LJTP2**-treated group (Fig. 4E). The above results indicated that the buffer and probe are nontoxic, stable, and specific for Hg detection without affecting plant health, root length, and overall plant growth.

### Distribution of Hg^2+^ in plants at the tissue level with single-photon microscopy imaging

Subsequently, confocal imaging was employed to investigate the plant tissue through the details of mercury–plant and mercury–plant–probe relationships.

#### Positive correlation of the fluorescence intensity of the probe with Hg^2+^ concentrations and incubation duration

**Fig. 5.**
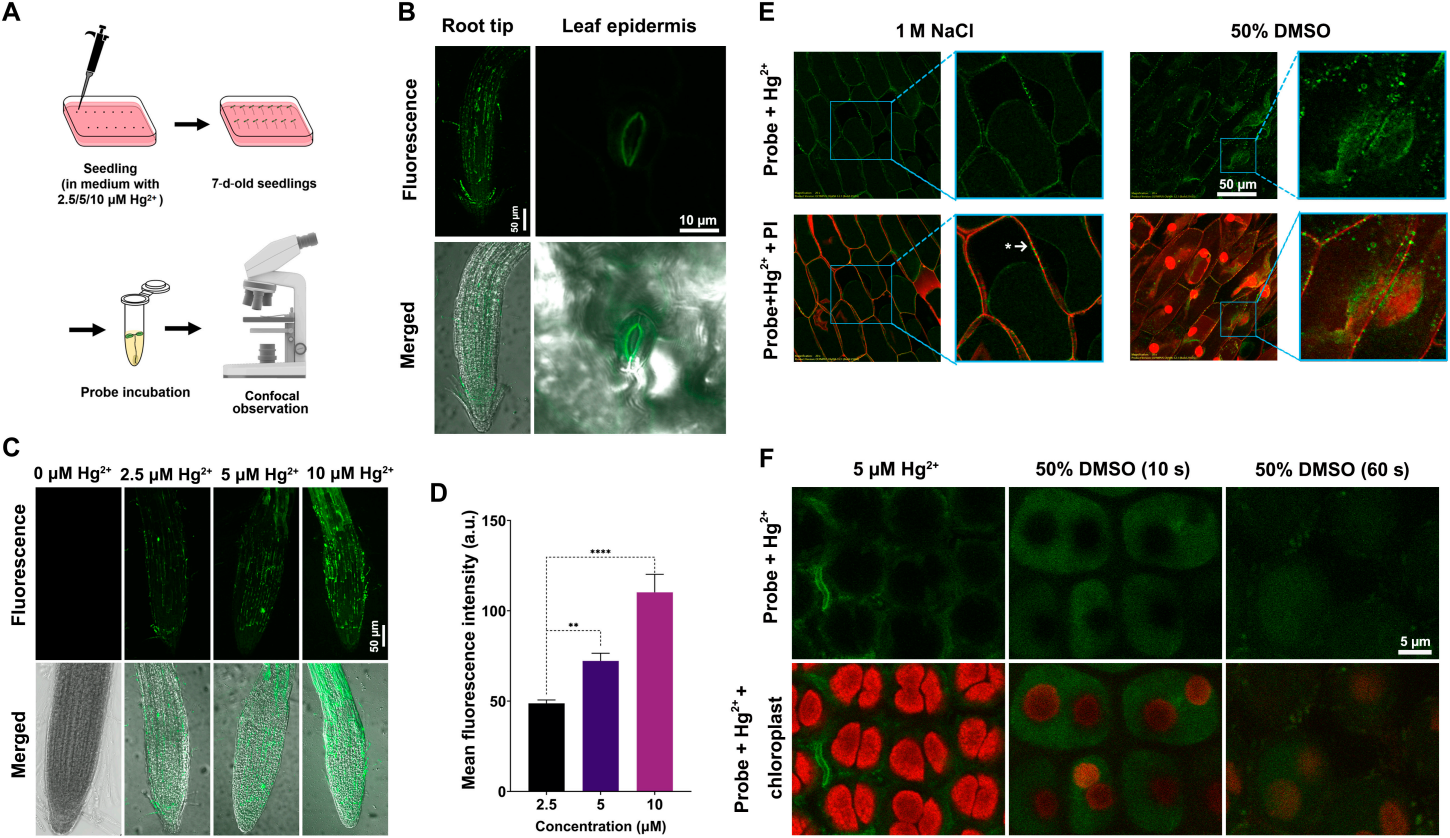
LJTP2 cell structure localization characteristics and mercury ion detection at the cellular and subcellular levels in *Arabidopsis thaliana*, onion, and moss using single-photon microscopy (*λ*_ex_ = 405 nm). (A) Schematic representation of the sample preparation process for single-photon microscopy. (B) Root tips and leaf epidermis of 5 μM Hg^2+^-treated *Arabidopsis* after 1 h of probe incubation. (C) Root tips of *Arabidopsis* treated with different concentrations of Hg^2+^ after 1 h of probe incubation. (D) Fluorescence quantification of the probe signal in the root tips of *Arabidopsis* treated with different concentrations of Hg^2+^ after 1 h of probe incubation. Statistical comparison was performed by one-sided *t* test. **P value ≤0.01; ****P value ≤0.0001. (E) Confocal microscopy images of Hg^2+^-treated onion epidermal cells, which were first treated with LJTP2 (10 μM) for a min and then incubated in 1.0 M NaCl and 50% dimethyl sulfoxide (DMSO) solutions, respectively. (F) Confocal microscopy images of Hg^2+^-treated moss leaf with or without DMSO. PI, propidium iodide.

To evaluate the growth environment under Hg^2+^ stress, the *A. thaliana* seedlings were incubated in 2.5, 5, and 10 μM Hg^2+^ media for 7 d and then stained with **LJTP2** (10 μM) for 1 h (Fig. 5A). The fluorescence signal was present in both root tip and leaf stomata regions, indicating Hg^2+^ distribution in *A. thaliana* (Fig. 5B). To further confirm Hg^2+^-induced fluorescence enhancement, the different concentrations were evaluated. The probe itself showed very weak fluorescence, but upon the addition of 2.5 μM Hg^2+^ into the media, the fluorescence signal was detected very clearly and became stronger with the increase in Hg^2+^ concentration (Fig. 5C). The mean fluorescence intensity statistical calculation is presented as well (Figure 5D). In addition, probe signals at different depths in *Arabidopsis* roots are shown in Figure S14. Overall, the results clearly indicated that the fluorescence intensity of the probe **LJTP2** was positively correlated with Hg^2+^ concentrations.

#### Strong affinity of LJTP2 to the plasma membrane validated in onion cells

To further study probe localization at the subcellular level, a plasmolysis experiment was performed in onion cells treated with NaCl and sucrose. When treated with NaCl, both showed a continuous probe signal at the cell membrane and a discontinued signal at the cell wall in plasmolysis analysis. Clear plasmolysis immediately took place after 3 to 4 min of Hg^2+^ treatment and 5 min of **LJTP2** incubation, followed by 20 min of NaCl (1.0 M concentration) (Fig. 5E). The plasma membrane was progressively detached from its neighboring cell wall, and the plasma membrane gradually shrank to an elliptical shape. Fluorescence signals were seen inside the cell in the cell nucleus when treated with Hg^2+^ solution for 40 min, followed by 1 min of 50% dimethyl sulfoxide (DMSO) (a dose of 50% was selected mainly because with the help of 50% DMSO, cell permeability was enhanced for Hg^2+^ penetration inside the cell) and 5 min of probe incubation respectively, as shown in (Fig. 5E). Therefore, the confocal imaging of **LJTP2** probe enabled monitoring of the morphological changes in the plasma membrane of the plant cell caused by the external environment under different physiological conditions.

#### LJTP2 unveils the selective permeability of the cell membrane structure toward Hg^2+^ in moss

To further confirm the location of **LJTP2** within the cell, the moss was sprayed with 5 μM Hg^2+^ 3 times in 3 d and then incubated with **LJTP2** for 1 h. As shown in Fig. 5F, the green fluorescence signals that **LJTP2** + Hg^2+^ induced were observed in the cell boundaries, and the reason is that the double-layer structure of the membrane blocked Hg intake/penetration at first; however, after treatment with DMSO for 10 s, the green fluorescence signal crossed the protoplast barrier and diffused all over the protoplasts except the chloroplast because the double-layer membrane structure of the chloroplast further stopped the entry of Hg^2+^ ions. Our results showed Hg^2+^ detection at the cell boundaries at first, and after the cell permeability was enhanced with DMSO treatment, Hg^2+^ further penetrated the chloroplast, so the probe entered all over the protoplast. Therefore, the fluorescence imaging in moss (another good model plant) reconfirmed the good sensing ability of **LJTP2** at the subcellular level.

### Subcellular localization and translocation of Hg^2+^ in plants detected by LJTP2 with 2-photon microscopy imaging

**Fig. 6.**
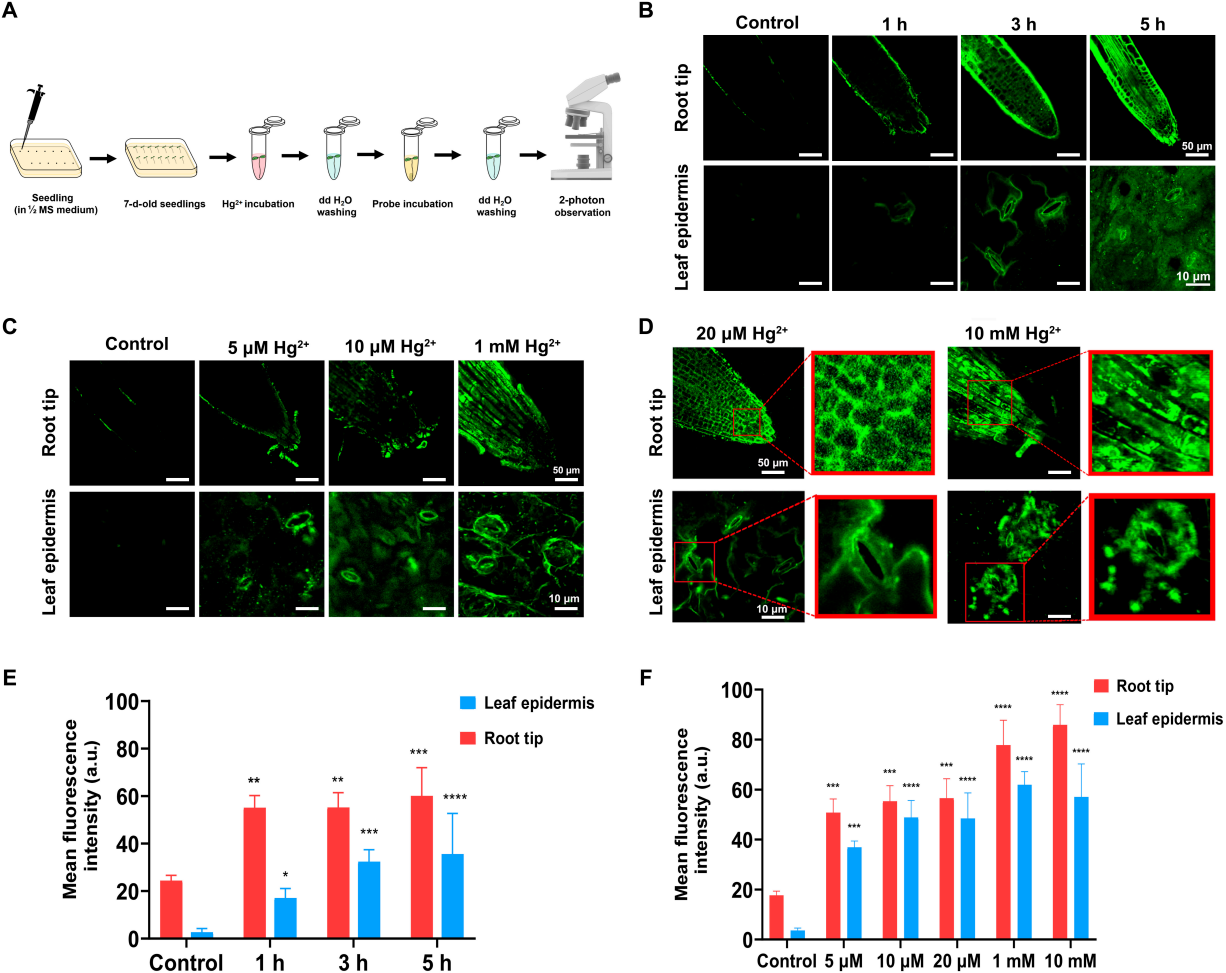
The subcellular localization of LJTP2 signals showed the translocation (progression) of Hg^2+^ in plant cells. (A) Schematic illustration of 2-photon imaging operation on *Arabidopsis* seedlings. (B) Two-photon imaging of a root tip at different time points after incubation of the probe (10 μM) with Hg^2+^ (100 μM). (C and D) 10 μM of probe concentration with Hg^2+^ (100 μM) showed the maximum translocation and strong signals on each cell within the root tip and leaf of *Arabidopsis* seedlings. (E and F) Calculated mean fluorescence intensities are presented. Statistical comparison was performed by a one-sided *t* test. *P value ≤0.05; **P value ≤0.01; ***P value ≤0.001; ****P value ≤0.0001. MS, Murashige and Skoog; dd H_2_O, double-distilled water.

Considering the advantages of 2-photon microscopy, including deeper penetration depth and reduced photodamage [21,22], 2-photon imaging was further conducted to reaffirm probe efficiency to detect Hg^2+^ at the subcellular level (Fig. 6).

#### LJTP2 revealed Hg^2+^ translocation progression and cell specificity through noninvasive localization of Hg^2+^ distribution in the subcellular structures of *A. thaliana*

The translocation of Hg^2+^ in plants and the stress effect of plant cells under Hg^2+^ were visualized using 2-photon microscopy (*λ*_ex_ = 750 nm) in a real-time manner (Fig. 6A). The probe detected a very faded signal, almost negligible, without Hg^2+^ incubation. When the root tips were incubated in Hg^2+^ solution and in the probe for 1 h, Hg^2+^ signals were detected all along the epidermis of the root tip. After 3 h of Hg^2+^ incubation, Hg^2+^ detection signals were all over the root tip at each cell. After 5 h of Hg^2+^ incubation followed by 1 h of the probe, there was a significant amount of Hg^2+^ translocation signals at the cell boundary of each cell (Fig. 6B). Consistent results were also observed in the leaf. The signals were not seen when the leaf samples were incubated without Hg^2+^ in the probe only. After 1 h of Hg^2+^ incubation, signals were observed at the leaf stomata, but after 5 h of Hg^2+^ incubation, more evident probe signals were observed in every cell. The Hg^2+^ incubation for different time points and probe soaking for a consistent time point indicated a positive and direct correlation of Hg^2+^ and the probe (Fig. 6B).

In summary, the fluorescence signal was located at the root epidermis after 1 h of probe incubation and then observed in root epidermal cells and also became stronger after 3- or 5-h incubation. Furthermore, the real-time imaging in leaves indicated that the Hg^2+^ translocation started from the stomata and moved to the vein and then the whole leaf. The Hg^2+^ distribution and translocation in the *A. thaliana* root and leaf were successfully observed spatiotemporally in a real-time manner under 2-photon imaging.

#### Spatiotemporal dynamic distribution of Hg^2+^ from epidermal cells to the root tip and leaf stomata shows a direct correlation of LJTP2 fluorescence intensity with Hg^2+^

To further investigate the status of cells under Hg^2+^ stress and signal progression, 2-photon imaging of *A. thaliana* roots and leaves at different concentrations of Hg^2+^ (Fig. 6C) showed the fluorescence signal in *A. thaliana* roots after incubation with 5 μM, which eventually got stronger with the increase in concentration from 5 μM to 1 mM Hg^2+^. The same trend was also observed in the leaves. Under low concentrations of Hg^2+^ treatment, the signal was distributed along the boundaries of plant root and leaf cells, whereas at high concentrations, mercury disrupted the integrity of the plant cell membrane, resulting in the signal filling the entire cell (Fig. 6D). The above results proved that **LJTP2** can be used to monitor the integrity of the cell structure under Hg^2+^ stress.

## Discussion

Hg^2+^ stress seriously affects agricultural and ecological safety; verifying the spatiotemporal distribution of Hg^2+^ in plant tissues and the accumulation-induced damage at the subcellular level is significant for the management of Hg pollution. Thus, there is an urgent need to develop an efficient imaging tool for monitoring Hg^2+^ stress in plant tissues. The fluorescent probe (**LJTP2**) was rationally designed with the amphiphilic nature of surfactants, which improved the probe’s dispersibility and sensitivity toward Hg^2+^ in aqueous solution. Compared with the key parameters and applications of some reported fluorescent probes for Hg^2+^, **LJTP2** displayed significant superiorities (Table S1), indicating the successful design of our probe.

Interestingly, the spatiotemporal dynamic distribution of Hg^2+^ in *A. thaliana* under 2-photon imaging revealed that the fluorescence signal initially appeared at the root exodermis within 5 min of probe incubation. The signal was further recorded in root epidermal cells and became stronger after 1 h of probe incubation. Real-time monitoring indicated that Hg^2+^ accumulation and distribution were mostly at the root. The real-time imaging in leaves indicated Hg^2+^ translocation from the stomata to the veins and then the whole leaf. Our findings also provided deeper insight, revealing that **LJTP2** showed a strong affinity toward cell structure boundaries in onion cells. The shrinkage of the cell protoplast led to a gap between the cell wall and cell membrane, which further resulted in discontinued signals at the cell wall but continued at the membrane as its structure remained intact. Furthermore, the Hg^2+^-induced damage on the cell membrane and stomata was clearly observed under 2-photon imaging, manifesting that **LJTP2** can be employed as an efficient visual tool to evaluate plant health in vivo.

In the future, **LJTP2** could be further developed to sense and detect Hg^2+^ presence fast and accurately to mitigate the problem of mercury contamination by combining the standard method phytoremediation removal of mercury from polluted areas through scavenger plants (a green strategy that uses hyperaccumulator plants and their rhizospheric microorganisms to transfer and degrade pollutants from the soil and environment)—with raising such plants by routine mutagenesis and genetic engineering [5,41].

## Conclusion

In summary, an amphiphilic fluorescent probe (**LJTP2**) was specifically designed to investigate the Hg^2+^ stress in plant tissues. **LJTP2** exhibited excellent selectivity and sensitivity for early Hg^2+^ detection in aqueous solution with a low LOD of 16 nM (Table S1). The plasmolysis experiment confirmed that **LJTP2** selectively targeted the cell membrane structure. Under confocal observations, the continuous and discontinuous distributions of Hg^2+^ in the cell membrane and cell wall were visualized obviously. Furthermore, the Hg^2+^ distribution and translocation and Hg^2+^ stress-induced membrane broken in the root tip cells and leaf stomata were well observed under 2-photon imaging. We believe this study not only provided a novel imaging tool for the investigation of Hg^2+^ stress on the plant cell structure but also facilitated the management of Hg pollution in agriculture.

## Materials and Methods

### Synthesis of the LJTP2 probe

**LJTP2** was synthesized using 4-bromo-1,8-naphthalic anhydride as a starting material, which further underwent nucleophilic, hydrolysis, condensation, and aminolysis reactions with moderate yields (Fig. 7). The nuclear magnetic resonance and high-resolution mass spectrometry data confirmed the chemical structure of **LJTP2**. The detailed synthetic procedure is depicted in the Supplementary Materials (Figs. S1 to S10).

**Fig. 7.**
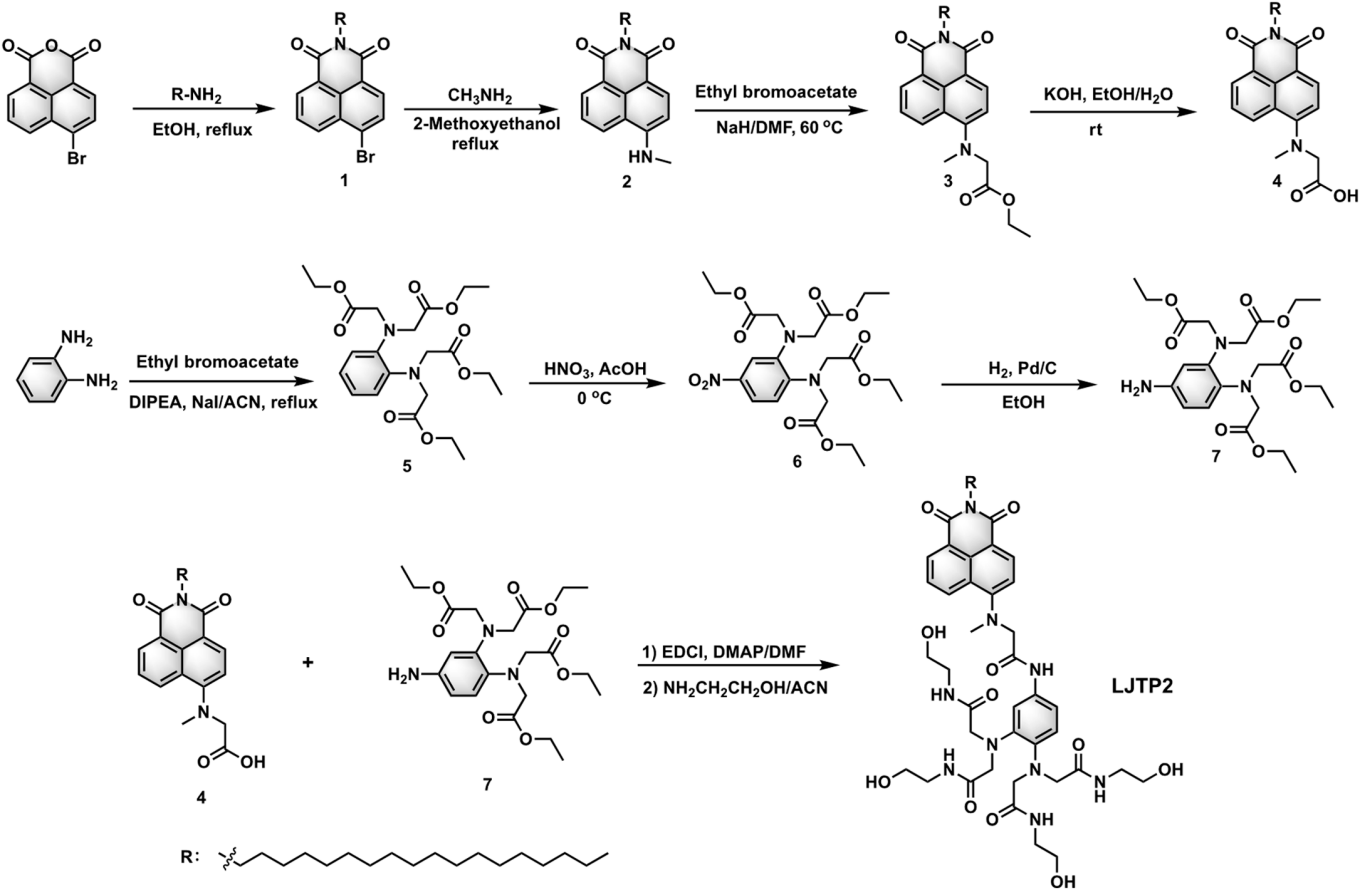
Synthetic route of the probe LJTP2. EtOH, ethyl alcohol; DMF, dimethylformamide; rt, room temperature; DIPEA, diisopropylethylamine; AcOH, acetic acid; EDCI, 1-ethyl-3-(3-dimethylaminopropyl)carbodiimide); DMAP, 4-dimethylaminopyridine.

### Plant material and growth conditions

*A. thaliana* was used. The plants were grown as control according to the protocol established by Sugimoto and Meyerowitz [42] and Chen et al. [43]. The seeds underwent a washing process involving a 10-min wash with 75% alcohol, followed by 4 successive washes with sterilized water; each step was kept 20 min. Subsequently, the washed seeds were refrigerated at 4 °C for 2 to 3 d before placing them on ½ Murashige and Skoog media. The medium was autoclaved at 121 °C for 21 min and later poured into petri dishes. After solidification, the seeds were sown, and plates were positioned vertically in a controlled environment growth chamber with conditions set at 22/20 °C for day and night with a 16-h photoperiod and illumination of approximately 500 lux from a white cool fluorescent lamp and maintained for 7 d. Moss, which were initially collected from the lion mountainous region of Wuhan, China, and subsequently grown in the laboratory, and onion were also used.

### Toxicity assessment of LJTP2

To evaluate the potential toxicity of the **LJTP2** probe on plants, 7-d-old *A. thaliana* was employed. Three treatments were established: the application of Hepes buffer, the application of the probe solution (10 μM in 40 mM Hepes buffer), and the application of distilled water, which was used as a control. Within each treatment, 4-time gradients were set: 30 min, 1 h, 12 h, and 24 h. The root length after treatment was measured as an index for assessing toxicity.

### Plant treatment and imaging

To explore the distribution of Hg^2+^ within the plant tissues, *A. thaliana* was grown on a medium containing HgCl_2_ (0, 2.5, 5, and 10 μM) for 7 d and then incubated in the **LJTP2** probe solution for 1 h. Subsequently, the samples were prepared into slides and imaged using an Olympus FV1000 confocal laser scanning microscope with an excitation wavelength of 405 nm.

The elemental selectivity test of the probe was conducted using *A. thaliana* as the material. Seven-day-old *Arabidopsis* seedlings were immersed in a solution containing 100 μM of 6 kinds of ions, namely, Hg^2+^, Cd^2+^, Zn^2+^, Mg^2+^, Na^+^, and K^+^, for 1 h. Distilled water treatment was used as a control. After treatment, the seedlings were rinsed with distilled water and incubated in the **LJTP2** probe solution for 1 h. The root signals were observed under a 2-photon mode on an Olympus FV1000 confocal microscope with an excitation wavelength of 750 nm.

The stability experiment of the probe was conducted using *A. thaliana* as the material. *A. thaliana* treated with 100 μM mercuric chloride and those treated with distilled water were processed in the **LJTP2** probe solution for different durations (5 min, 1 h, 12 h, and 24 h). The signals were observed under an Olympus FV1000 single-photon confocal microscope with an excitation wavelength of 405 nm.

To explore the dynamics of Hg^2+^ entry into plant cells and the cellular-level distribution of mercury ions within plants. *A. thaliana* was grown on a culture medium for 7 d and then immersed in a solution of 100 μM mercuric chloride for different durations (1, 3, and 5 h) or in solutions of varying concentrations (5 μM, 10 μM, 20 μM, 1 mM, and 10 mM) of HgCl_2_ for 2 h. The signals from the root tips and leaf epidermis were observed using the 2-photon mode of an Olympus FV1000 confocal microscope with an excitation wavelength of 750 nm.

### Plasmolysis experiments in onions

Onion epidermis tissues were cut with the help of tweezers and scissors [44]. These tissues were first incubated in Hg^2+^ solution for 40 min and later dipped for 1 min in probe solution. Then, the plasmolysis experiments were performed with NaCl (1 mol/l). Then, DMSO (50% concentration) treatment for 1 s enhanced the permeability of the cell membrane of the onion tissues to examine the probe’s affinity to the membrane

### Measurement of quantitative data and statistical analysis

Morphological data, including root length and fluorescence intensities, were calculated using the ImageJ software. Subsequently, data were statistically analyzed, the means and standard deviation were calculated, and the analysis of variance test was applied to check the significance levels. Statistical analyses and graphs were performed and generated using the GraphPad Prism software, respectively.

## Data Availability

There are no restrictions on data availability.
