# Supplementary material for: Visualization of Hg2+ Stress on Plant Health at the Subcellular Level Revealed by a Highly Sensitive Fluorescent Sensor
Source: Research (Wash D C). 2025 Jan 7;8:0570. doi: 10.34133/research.0570 (PMC11704093; doi:10.34133/research.0570)
Supplement: Supplementary 1 — Supplementary Data Figs. S1 to S14 Table S1 [file research.0570.f1.docx]

Supplementary data

Compounds 5, 6 and 7 as shown in Figure 1, were synthesized according to the literature (*Analyst*, 2012, 137, 1837-1845).

Synthesis of compound 1

Octadecyl amine (12 mmol, 3.3 g) and 4-Bromo-1,8-naphthalic anhydride (10 mmol, 2.8 g) were dissolved in 30 mL EtOH and refluxed for 24 hours. After cooling down to room temperature, the precipitated solids were filtered and washed with hexane to afford the crude **compound 1** as grey solid 6.0 g which can be directly used in next step.

Synthesis of compound 2

**Compound 1** (11.3 mmol, 6.0 g) and 33% methylamine in MeOH (60 mmol, 7.3 mL) were dissolved in 50 mL 2-Methoxyethanol. The mixture was heated at 90 ^o^C for 24 hours. After completing the reaction, the solvent was removed under reduced pressure and the residue was purified via column chromatography (CC) to get **compound 2** as yellow solid 2.0 g. ^1^H NMR (400 MHZ, CDCl_3_) δ: 8.59 (d, *J* = 7.2 Hz, 1H), 8.49 (d, *J* = 8.0 Hz, 1H), 7.62 (t, *J* = 8.0 Hz, 1H), 6.72 (d, *J* = 8.4 Hz, 1H), 5.35 (s, 1H), 4.15 (t, *J* = 8.0 Hz, 2H), 3.15 (s, 3H), 1.76 - 1.72 (m, 3H), 1.41 - 1.24 (m, 32 H), 0.88 (t, *J* = 6.4 Hz, 3H). ^13^C NMR (100 MHZ, CDCl_3_) δ: 164.7, 164.2, 150.4, 134.4, 131.0, 129.7, 126.0, 124.6, 123.1, 120.3, 110.3, 103.8, 40.3, 31.9, 30.5, 29.7 – 29.6 (m), 29.5, 29.4, 28.3, 27.2, 22.7, 14.1. HRMS calcd for [M + Na]^+^: 501.3451, found: 501.3441.

Synthesis of compound 3

**Compound 2** (1 mmol, 478 mg) and Ethyl bromoacetate (2 mmol, 334 mg) and NaH (3 mmol, 72 mg) were dissolved in 10 mL anhydrous DMF. After heating at 65 ^o^C overnight, the solvent was removed under reduced pressure, the residue was dissolved in CH_2_Cl_2_, and washed by water and saturated NaCl, the organic layer was separated and dried by anhydrous Na_2_SO_4_. After evaporating CH_2_Cl_2_, the residue was purified via CC to afford compound 3 as yellow solid 0.18 g. ^1^H NMR (400 MHZ, CDCl_3_) δ: 8.51 (dd, *J* = 7.2, 1.2 Hz, 1H), 8.42 (d, *J* = 8.0 Hz, 1H), 8.35 – 8.32 (dd, *J* = 8.4, 1.2 Hz, 1H), 7.62 – 7.58 (m, 1H), 7.19 (t, *J* = 5.2 Hz, 1H), 4.19 – 4.15 (m, 3H), 4.09 – 4.03 (m, 4H), 3.09 (s, 3H), 1.65 – 1.62 (m, 2H), 1.35 – 1.15 (m, 32 H), 0.80 (t, *J* = 6.8 Hz, 3 H). ^13^C NMR (100 MHZ, CDCl_3_) δ: 170.0, 164.5, 164.0, 155.0, 132.2, 131.1, 130.3, 130.1, 125.6, 125.5, 123.4, 116.4, 115.5, 68.2, 61.3, 58.6, 41.5, 40.4, 31.9, 29.7 – 29.4 (m), 28.2, 27.2, 22.7, 14.2. HRMS calcd for [M + H]^+^: 565.4000, found: 565.3994.

Synthesis of compound 4

**Compound 3** (0.53 mmol, 300 mg) were dissolved in EtOH/H_2_O (5:1, 6 mL), KOH (1.2 mmol, 68 mg) was added to above mixture. The reaction was stirred at room temperature for 24 hours. After completing the reaction, the pH was adjusted to 3.0 via addition of 2N HCl. The precipitated solids were washed with water and hexane respectively to get the crude product 220 mg which can be directly used for the next step. ^1^H NMR (400 MHZ, CDCl_3_) δ: 8.49 (d, *J* = 6.4 Hz, 1H), 8.42 – 8.39 (m, 1H), 8.30 (d, *J* = 8.0 Hz, 1H), 7.60 – 7.59 (m, 1H), 7.20 (d, *J* = 6.0 Hz, 1H), 4.08 (s, 2H), 3.07 (s, 3H), 1.63 – 1.62 (m, 2H), 1.35 – 1.21 (m, 32 H), 0.80 (t, *J* = 6.8 Hz, 3H). HRMS calcd for [M + H]^+^: 537.3687, found : 537.3685.

Synthesis of **LJTP2**

**Compound 4** (0.41 mmol, 220 mg), EDCI (0.8 mmol, 153 mg) and DMAP (0.1 mmol, 12 mg) were dissolved in 8 mL anhydrous DMF and stirred for 1hour. **Compound 7** (0.41 mmol, 192 mg) were added to above mixture and kept stirring at room temperature for 12 hours. The solvent was removed under reduced pressure, and the resulting residue was purified via CC to get the condensation product as yellow solid 215 mg which reacted with ethanolamine (6 mL) in 10 mL ACN solution at 80 ^o^C for 8 hours. After completing the reaction, the mixture was washed with saturated NaCl and extracted by CH_2_Cl_2_. The organic layer was purified via CC to get **LJTP2** as yellow oil 98 mg. ^1^H NMR (600 MHZ, CDOD) δ: 8.56 (d, *J* = 8.4 Hz, 1H), 8.43 (d, J = 7.2 Hz, 1H), 8.37 – 8.35 (m, 1H), 7.42 (s, 1H), 7.29 (d, *J* = 7.8 Hz, 1H), 7.17 – 7.15 (dd, *J* = 9.0, 2.4 Hz, 1H), 7.00 (d, *J* = 9.0 Hz, 1H), 4.62 (s, 6H), 4.15 – 4.06 (m, 12 H), 3.75 (t, J = 5.4 Hz, 2H), 3.51 – 3.49 (m, 8H), 3.27 – 3.24 (m, 8H), 3.14 (s, 3H), 3.03 (t, *J* = 4.8 Hz, 2H), 1.67 – 1.64 (m, 2H), 1.36 – 1.26 (m, 32H), 0.88 (t, J = 7.2 Hz, 3H). HRMS called for [M + Na]^+^: 1068.6104, found : 1068. 6102.


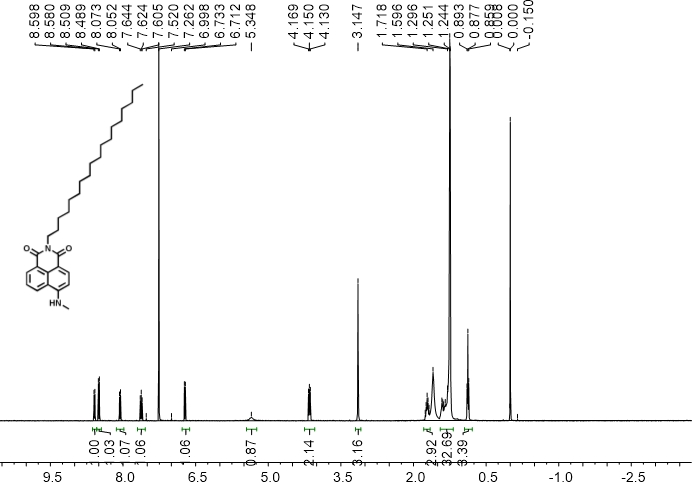


**Figure S1.** ^1^H NMR spectrum of **compound 2.**


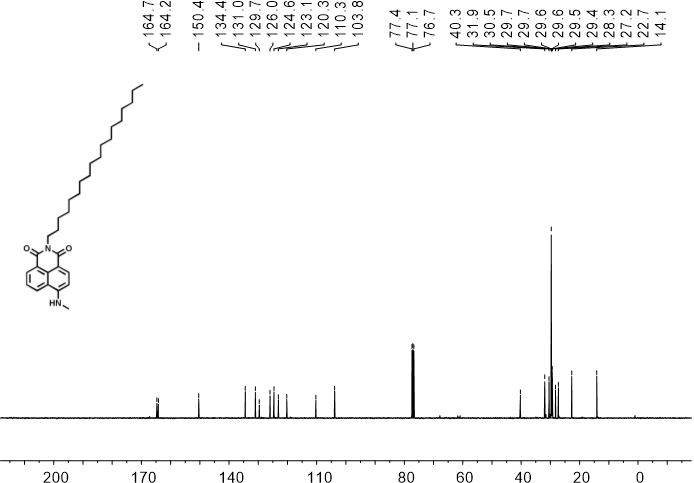


**Figure S2.** ^13^C NMR spectrum of **compound 2.**


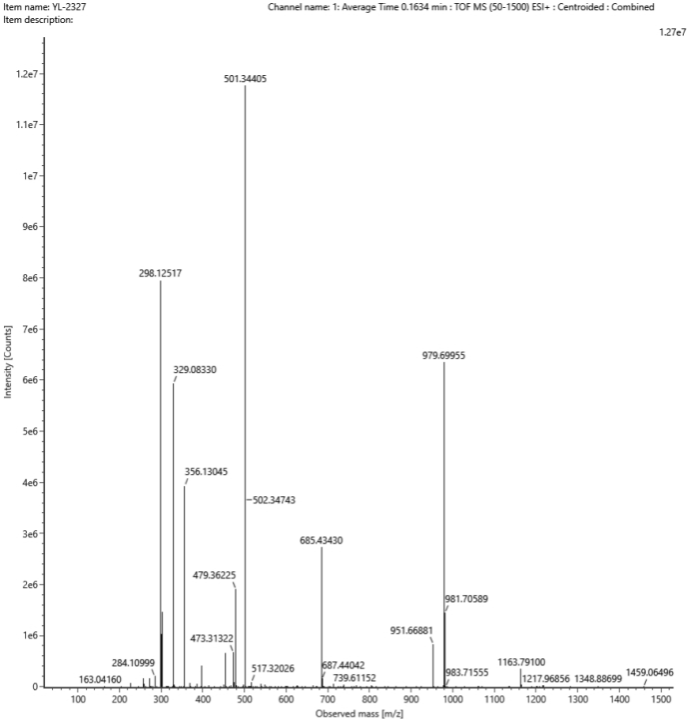


**Figure S3.** HRMS spectrum of **compound 2.**


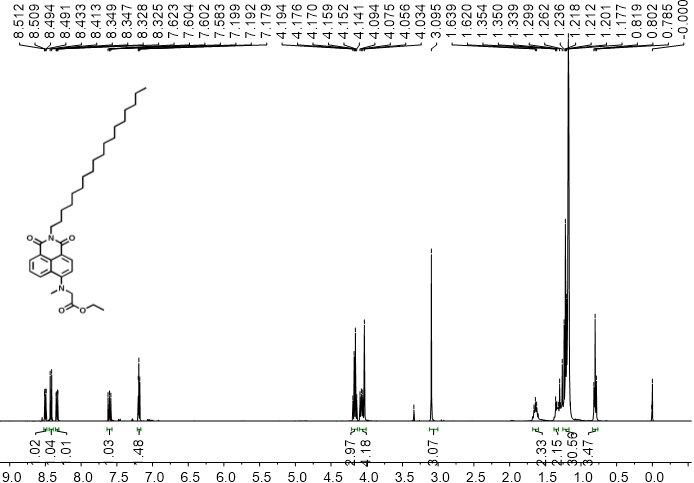


**Figure S4.** ^1^H NMR spectrum of **compound 3.**


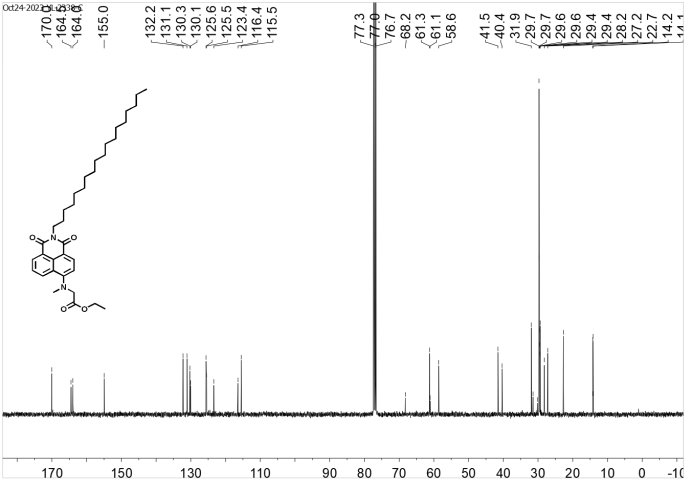


**Figure S5.** ^13^C NMR spectrum of **compound 3.**


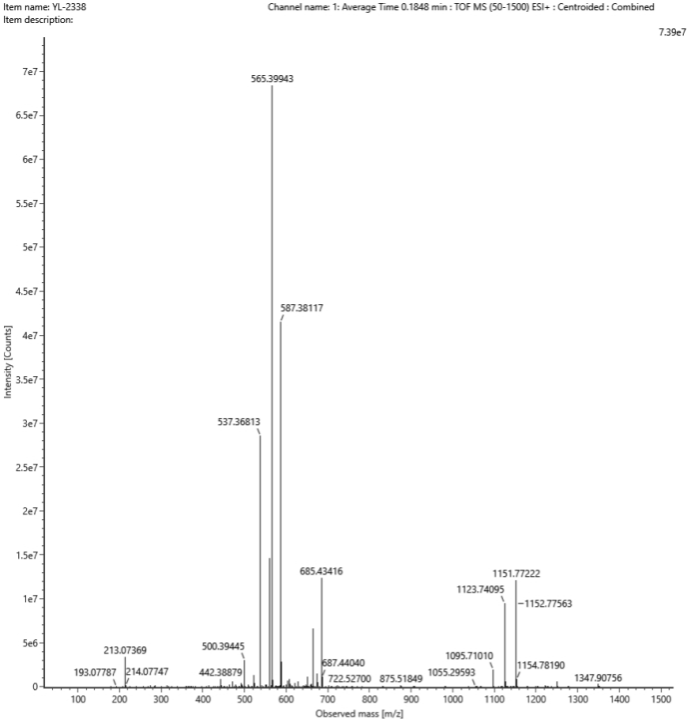


**Figure S6.** HRMS spectrum of **compound 3.**


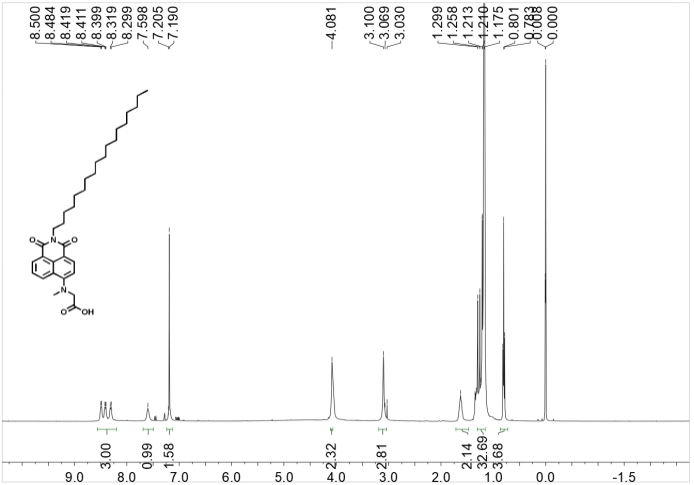


**Figure S7.** ^1^H NMR spectrum of **compound 4.**


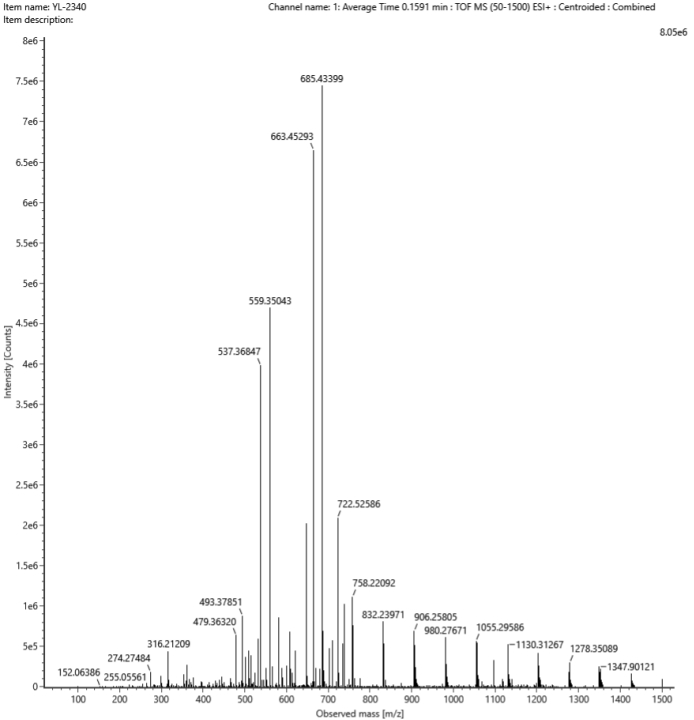


**Figure S8.** HRMS spectrum of **compound 4.**


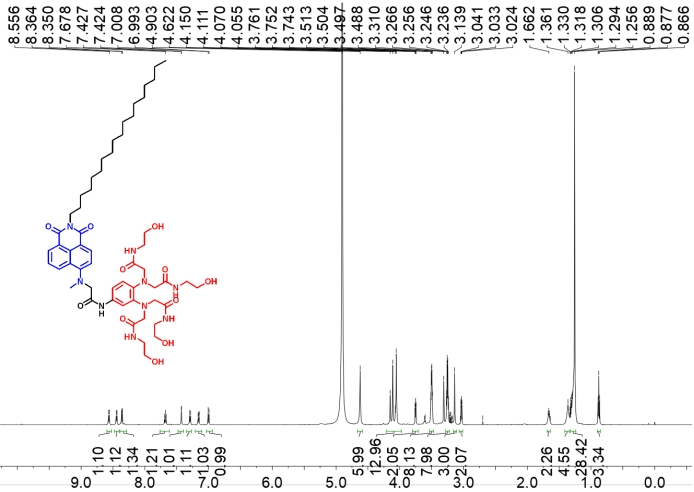


**Figure S9.** ^1^H NMR spectrum of **LJTP2.**


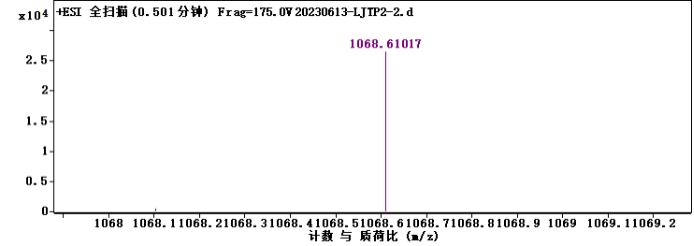


**Figure S10.** HRMS spectrum of **LJTP2.**


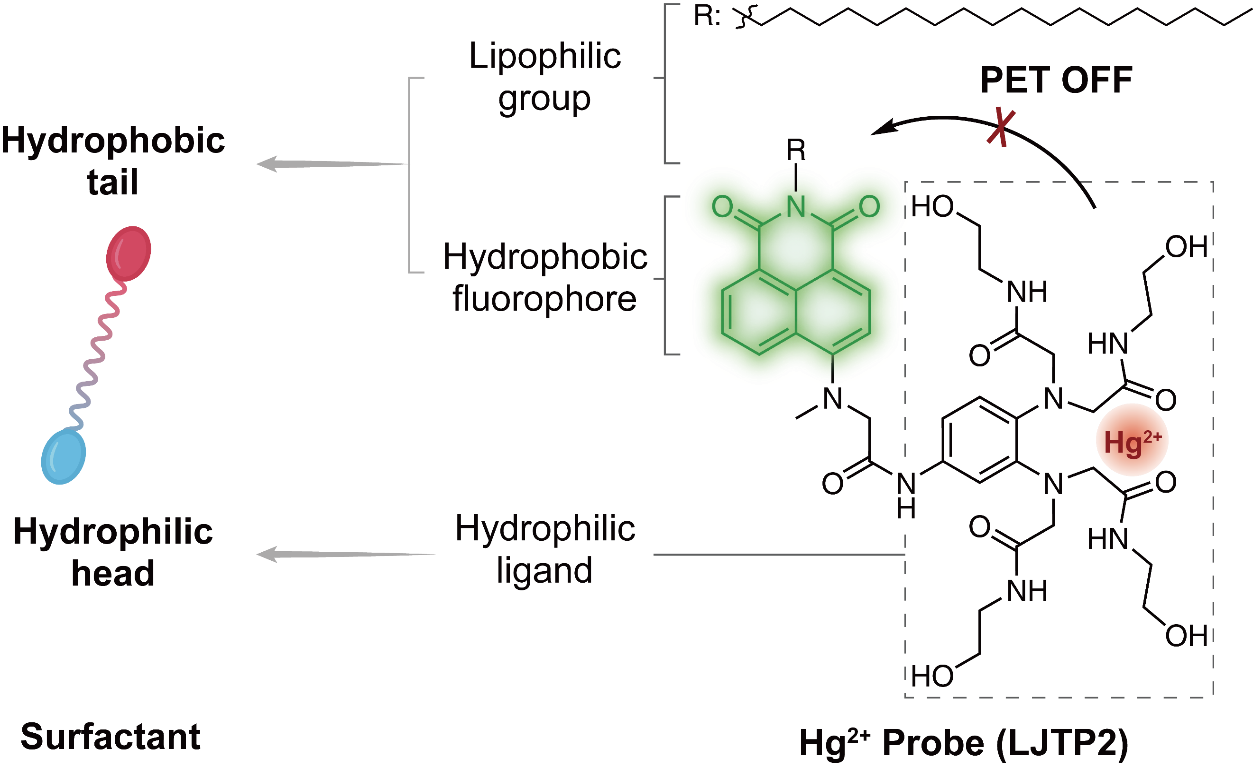


**Figure S11.** The sensing mechanism of LJTP2 towards Hg^2+^


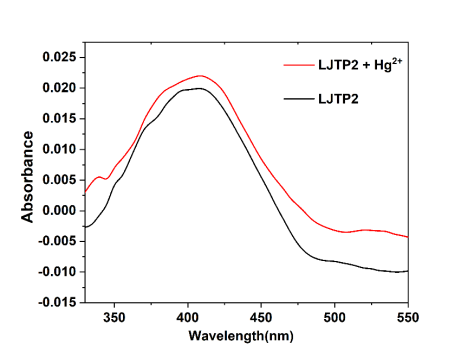


**Figure S12.** The UV spectrum of **LJTP2** with (black line) and without (red line) of Hg^2+^

**Figure S13.** pH measurement against fluorescent intensity of probe and probe with Hg^2+^.

**Table S1** Representative fluorescent probes for the detection of Hg^2+^

| **Structure** | **Solvent** | **LOD**  **(μM)** | **Imaging applications** | **Reference** |
| --- | --- | --- | --- | --- |
|  | DMF : H_2_O  = 3:7 | 266 | Living cells, zebrafish, mice and *A. thaliana* | Dyes Pigm.  2022, 200, 100. |
|  | Na_2_HPO_4_ and citric acid buffer | 0.0168 | Cells and zebrafish. | J. Hazard.  Mater.  2022, 424, 127701. |
|  | PBS : CH_3_CN  = 1:1 | 0.04 | Mouse | J. Hazard. Mater.  2023, 445, 130612. |
|  | PBS : DMF  = 9:1 | 0.63 | Living cells,  onion and zebrafish | J. Hazard. Mater.  2024, 461, 132604. |
|  | PBS : CH_3_CN  = 9:1 | 0.35 | HeLa cells | J. Hazard. Mater.  2024, 464, 132999. |
|  | CH_3_CN : H_2_O  = 2 : 3 | 0.3 (ppb) | HeLa cells | Chem. Sci.  2017, 8, 2047. |
|  | PBS | 0.027 | HeLa cells and E. coli. | Chem. Commun.  2018, 54, 4955. |
|  | HEPES | 0.043 | Live cells and tissues | Chem. Commun.  2019,  55, 1766. |
| 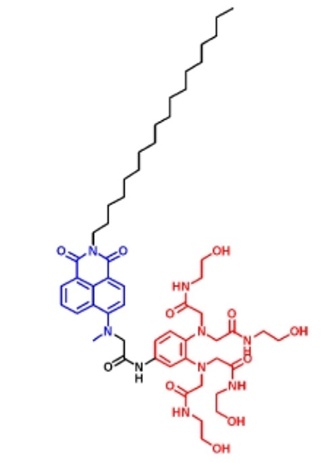 | HEPES | 0.016 | *A. thaliana*, onion and moss | **This work** |


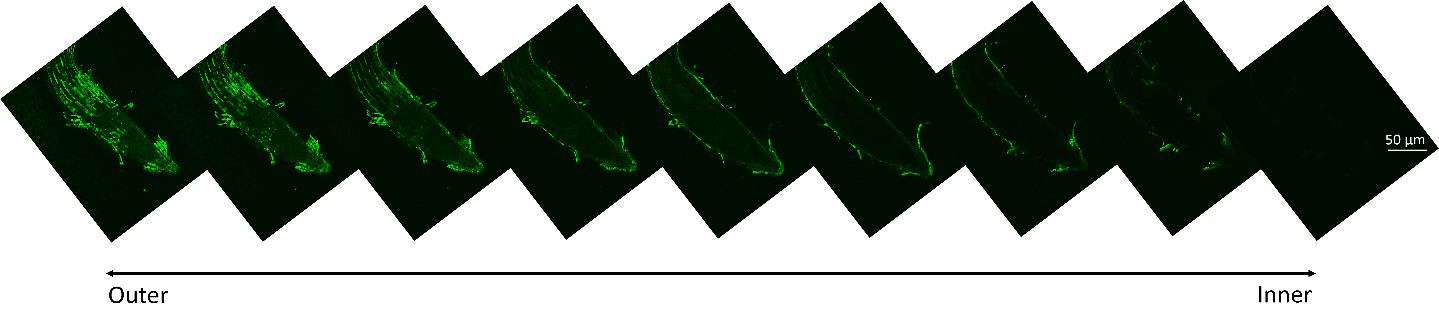


**Figure S14**. Fluorescence images taken at different depths revealed the spatial distribution of probe signals in Arabidopsis root tips.
